# Demographic and socioeconomic inequalities in ideal cardiovascular health: A systematic review and meta-analysis

**DOI:** 10.1371/journal.pone.0255959

**Published:** 2021-08-11

**Authors:** Janko Janković, Stefan Mandić-Rajčević, Maša Davidović, Slavenka Janković

**Affiliations:** 1 Institute of Social Medicine, Faculty of Medicine, University of Belgrade, Belgrade, Serbia; 2 Graduate School for Health Sciences, University of Bern, Bern, Switzerland; 3 Institute of Epidemiology, Faculty of Medicine, University of Belgrade, Belgrade, Serbia; Queensland University of Technology, AUSTRALIA

## Abstract

**Background:**

In 2010, the American Heart Association introduced a new concept of ideal cardiovascular health (CVH) defined as the simultaneous presence of 7 favorable CVH metrics (smoking, diet, physical activity, body mass index, blood pressure, total cholesterol, and fasting blood glucose). The objective of this study was to conduct a systematic literature review and meta-analysis of studies examining the prevalence of ideal CVH, and each of the ideal CVH metrics as well as the relationship between socio-demographic determinants and ideal CVH.

**Methods:**

A comprehensive literature search was conducted in Medline and Scopus databases for studies published between 1 January 2010 and 30 June 2020. A total of 50 studies including 2,148,470 participants were analyzed. Associations were estimated using DerSimonian-Laird random-effect models. Heterogeneity was investigated through subgroup analyses, *Q*-test, and *I*^*2*^ statistics.

**Results:**

This study showed a low prevalence of ideal CVH defining as 6 and 7 ideal metrics (3.3%). Among seven ideal CVH metrics, smoking was the best metric (71%), while the poorest CVH metric was a healthy diet (5.8%). Gender was a statistically significant moderator of ideal smoking (81% in females and 60% in males) and ideal blood pressure (42% in females and 30% in males). Females and young adults had better CVH status compared to males and older adults. Also, more educated and better-off individuals had a greater number of ideal CVH metrics.

**Conclusions:**

To the best of our knowledge, this is the first systematic review on the relationship between participants’ socioeconomic status and ideal CVH. The results suggest that the prevalence of ideal CVH and most metrics was unsatisfactory. In order to achieve the improvement of the CVH metrics and the overall ideal CVH, nationwide prevention efforts at the population and individual levels are urgently needed.

## Introduction

In 2010, the American Heart Association (AHA) introduced a new concept of ideal cardiovascular health (CVH) as part of its efforts to improve the CVH of all Americans while reducing deaths from cardiovascular disease (CVD) [1]. This concept is defined as the simultaneous presence of 7 favorable CVH metrics or “Life’s Simple 7”: 4 health behaviors (smoking, diet, physical activity, and body mass index) and 3 health factors (blood pressure, total cholesterol, and fasting blood glucose) in the absence of CVD.

Since that time, many population-based studies have examined the prevalence of AHA’s ideal CVH, ideal CVH metrics, and their distribution by socio-demographic characteristics [2–10]. To examine associations between ideal CVH metrics and CVD events and non-CVD endpoints, several prospective cohort studies [2, 11–14], systematic review and meta-analyses [15–18] were conducted. Their results showed that ideal CVH metrics are inversely associated with the risk of CVD events [2, 13, 14, 16, 17], and both all-cause and CVD-related mortality [11–14, 16–18]. Studies suggested that ideal CVH status and even modest improvements in CVH metrics are beneficial for substantial reductions in the risk of CVD events [17] and CVD-related mortality [17, 18].

Therefore, evaluating associations between socio-demographic characteristics and ideal CVH metrics would be a valuable resource for communities to develop public health and clinical interventions and policies to improve ideal CVH and consequently prevent CVD events. However, studies that synthesize data on the prevalence of 7 ideal CVH metrics, ideal CVH, and their distribution by socio-demographic characteristics are lacking. To the best of our knowledge, there is only one systematic review [15] and one meta-analysis [19] on this topic. Accordingly, we conducted a comprehensive systematic review and meta-analysis with the aim to synthesize data on the prevalence of ideal CVH, and each of the ideal CVH metrics. What is new in our review compared to the previous reviews, is the updating of the population’s ideal CVH due to the significant number of recently published studies, and the fact that we examined the relationship between social determinants such as education and income and ideal CVH.

## Method

### Search strategy for identification of studies

We performed a comprehensive electronic search of published studies from their inception to June 2020 by searching Medline (via PubMed) and Scopus databases with the following terms: "cardiovascular health", "Life’s simple 7", and "ideal" in English. The search string used for the review in PubMed was: ((cardiovascular health[Title]) OR (Life’s simple 7[Title])) AND (ideal) AND ("2010"[Date—Publication]: "2020/06/30"[Date—Publication]) AND (English[Language]), while for Scopus: (TITLE ("cardiovascular health") OR TITLE ("Life’s simple 7")) AND ALL (ideal) AND LANGUAGE (English) AND PUBYEAR > 2009.

To identify any articles missed through the database search, we manually reviewed the reference lists of the selected studies.

### Data collection and analysis

#### Selection of studies

As a first step of the study selection process, two members of the research team (MD and JJ) screened independently the titles and abstracts of the identified articles and excluded duplicates and articles not relevant to the topic. The process between two researchers was compared, and any uncertainties were discussed and solved by the third researcher (SJ). If any key information was missing, we contacted the study authors to provide additional information. If this was not possible or ineffective, the study was rejected.

We documented the study selection process in a Preferred Reporting Items for Systematic Reviews and Meta-Analyses (PRISMA) flow chart [20].

#### Eligibility criteria

Articles were eligible for inclusion if written in English, peer-reviewed, and published between January 1, 2010 and June 30, 2020. To be included in the review process, studies had to assess the prevalence of ideal CVH metrics (smoking, physical activity, healthy diet, BMI, blood pressure, total cholesterol, and fasting blood glucose) as defined by the AHA [1]. The AHA criteria for the definition of ideal, intermediate, and poor CVH metrics are presented in S1 Table. Furthermore, studies were included if they enrolled participants 18 years or older who were free of CVD. Review articles, meta-analyses, commentaries and discussions, editorials, letters to editors (except when all relevant data was available), conference papers, books or book chapters were excluded.

#### Data extraction and quality assessment

Two review authors (MD and JJ) independently extracted and entered data from all included studies into the Characteristics of included studies table. A third review author (SJ) checked the extracted data. The information included the following: first author and year of publication; country where the study was conducted; study design; study date; number of participants enrolled; percentage of male; age/range and mean years (SD) of participants; and main outcome measures. The main outcome measures of this review are prevalence (%) and 95% confidence intervals (CI) of ideal CVH metrics and ideal CVH, ideal health behaviors and ideal health factors, odds ratio (OR) and 95% CI, prevalence ratio (PR) and 95% CI or relative predicted score differences (RPS) and 95% CI of having ideal CVH.

For studies with more than one publication, we considered the first publication as the primary reference.

The quality of each study was evaluated using a standardized 14-item National Institutes of Health Quality Assessment Tool for Observational Cohort and Cross-Sectional Studies (NIH-QAT) [21]. Each study was rated as good (most methodological criteria met, low risk of bias), fair (some criteria met, low risk of bias), or poor (few criteria met, high risk of bias).

Since almost half of the NIH-QAT items are not applicable to the studies included in our analysis, we additionally used the risk of bias tool specifically designed for prevalence studies [22]. It consists of 10 items addressing four domains of bias plus a summary risk of bias assessment. The response options for each item were either yes or no. Studies with yes answered for 0 to 3, 4 to 6, and 7 to 10 items were regarded as having overall high, moderate and low risk of bias, respectively.

Two researchers (JJ and SJ) independently appraised each study meeting inclusion criteria and subsequently, disagreements were discussed and solved by consensus on the final rating of the quality for each study. The reliability of the quality assessment between researchers was calculated using the kappa (k) statistic.

Our study followed all PRISMA guidelines, as applicable, in the design, data collection, analysis and reporting of this systematic review and meta-analysis (S2 Table).

#### Statistical analysis

The meta-analysis was carried out using the proportion or the double arcsine transformed proportion (in the case of proportions between 0 and 0.2) as the outcome measure. Both a fixed and random-effects model were fitted to the data and the amount of heterogeneity (i.e., *τ*^2^) was estimated using the DerSimonian-Laird estimator [23]. In addition, the *Q*-test for heterogeneity [24] and the *I*^2^ statistic [25] were reported. Studentized residuals and Cook’s distances were used to examine whether studies may be outliers and/or influential in the context of the model [26]. In subgroup analysis, for all ideal CVH metrics, and the presence of 5, 6, and 7 ideal CVH metrics and for ideal CVH, proportions were stratified into two groups based on gender (female and male). For ideal CVH proportions were stratified by age into old, middle, and young groups. Mixed-effects models were used to test whether the proportions across these subgroups vary significantly from each other, and the Q (QM) statistic was used to check whether the two or three groups have significantly different outcomes [25]. The analysis was carried out using R Programming Language and Environment for Statistical Computing (version 4.0.2) [27] and the metafor package (version 2.4.0) [28].

This review was registered in the PROSPERO (CRD42020152644) on 28 April 2020.

## Results

### Description of studies

#### Results of the search

**Fig 1:**
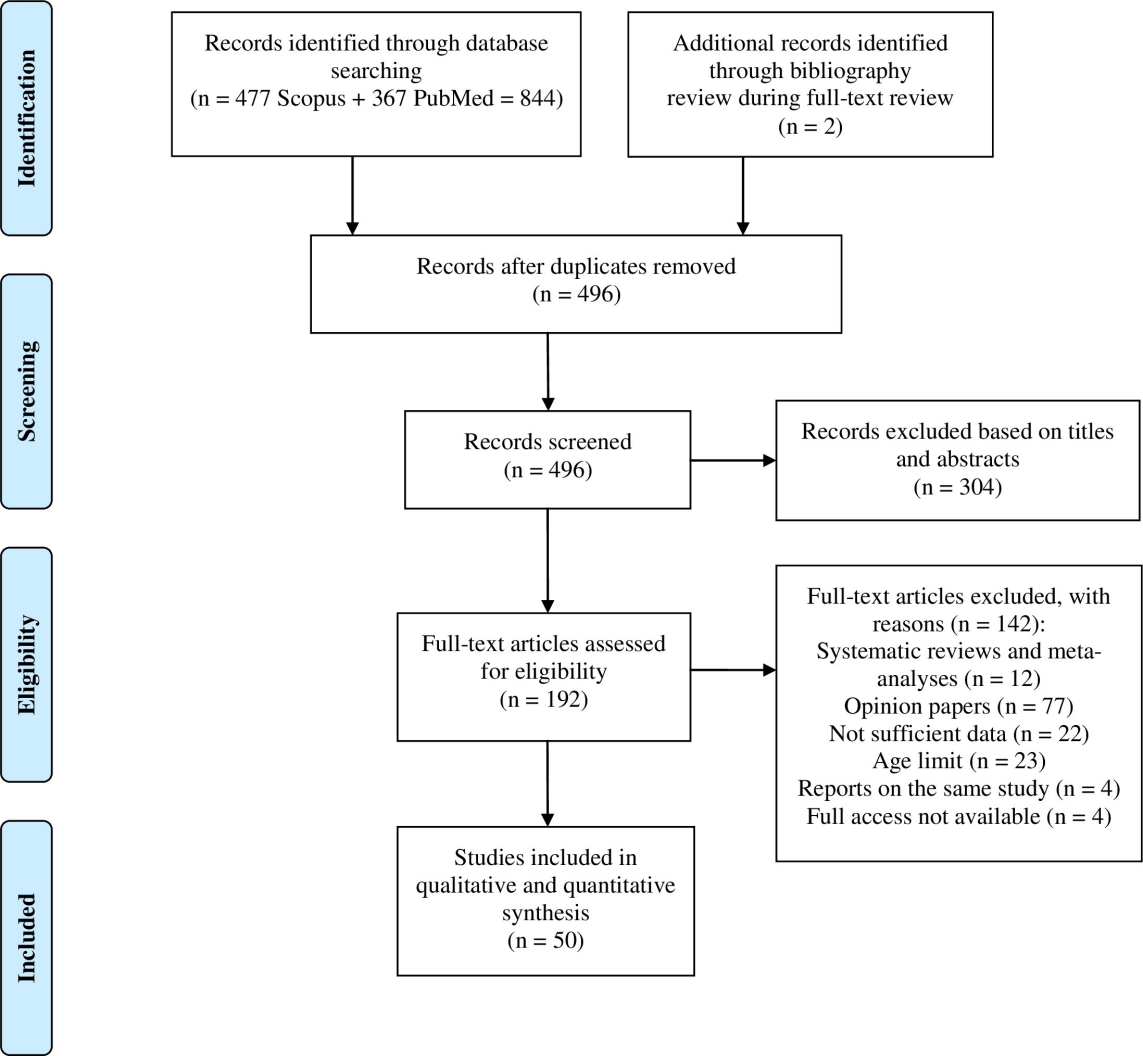
Study flow diagram. A PRISMA flow diagram that details the inclusion and exclusion of studies considered for this systematic review. PRISMA, Preferred Reporting Items for Systematic Reviews and Meta-Analyses.

The initial database search yielded 844 records, and two records were obtained from other sources (reference lists of articles identified through database searching). We screened the titles and abstracts of a total of 496 non-duplicate records and excluded 304 articles not relevant to the topic. A total of 192 full-text articles were reviewed for eligibility, and 50 studies that met our search criteria were included in the analysis. Detailed results of our search are presented in Fig 1 as a PRISMA flowchart.

#### Characteristics of included studies

All 50 studies [2, 4, 8–10, 29–73] included in the review were primarily cross-sectional or cross-sectional nested in a longitudinal study (Table 1). Of all included studies, 17 originated from the USA, 11 from China (one of them from Hong Kong), three from Brazil, two from France, one from Latin America (Argentina, Chile, Uruguay), and one study from each of the following countries: Canada, Australia, Nepal, Korea, Iran, Peru, Venezuela, Ecuador, India, Ghana, Spain, Finland, BH (Republic of Srpska), Serbia, Czech Republic, and Poland (Table 1).

**Table 1:** Characteristics of included studies.

First author, year	Country	Study design	Study date	No of population (% male)	Age range; mean age (SD)	Main outcome measures
Bambs CE et al. 2011 [29]	USA	Cross-sectional study	Heart SCORE study 2003	1933 (34%)	45–75; 59 (7.5)	Prevalence of ideal CVH, CVH metrics, ideal health behaviors, and health factors
Benziger CP et al. 2018 [30]	Peru	Cross-sectional study	CHRONICAS 2010	3058 (48.7%)	≥35; 55.6 (12.7)	Prevalence of ideal CVH and CVH metrics, PRs of ideal CVH for education and Wealth index
Bi Y et al. 2015 [31]	China	Cross-sectional study	2010	96121 (45.7%)	≥20	Prevalence of ideal CVH and CVH metrics, ideal health behaviors, and health factors
Bundy JD et al. 2020 [32]	USA	Cross-sectional nested in cohort study	1985–2016	30447 (39.4%)	55.0 (13.9)	Prevalence of ideal CVH and CVH metrics
Chang Y et al. 2016 [33]	China	Cross-sectional study	2012–2013	11113 (46.2%)	≥35; 53.8 (10.6)	Prevalence of ideal CVH, CVH metrics, ideal health behaviors, and health factors, ORs of ideal CVH for education and family income
Chung JWY et al. 2018 [34]	Hong Kong, China	Cross-sectional study	2014–2016	626 (9.2%)	>20	Prevalence of ideal CVH and CVH metrics
De Moraes ACF et al. 2019 [10]	USA	Cross-sectional study	2000–2002	6792 (47.2%)	45–84; 62.2	Prevalence of ideal CVH and CVH metrics
Del Brutto OH et al. 2013 [35]	Ecuador	Cross-sectional study	Atahualpa 2012;	616 (40.6%)	40–99; 59.0 (13.0)	Prevalence of ideal CVH and CVH metrics
	USA		NOMAS 1993–2001	1617 (36.7%)	40–107; 66.0 (9.0)	
Djousse L et al. 2015 [36]	USA	Cross-sectional nested in cohort study	JHS 2000–2004 (baseline visit)	5301 (36.5%)	55.3 (12.7)	Prevalence of ideal CVH metrics, ideal health behaviors, and health factors, and number of ideal CVH metrics
Fan C et al. 2020 [37]	USA	Cross-sectional nested in cohort study	APAC 2012	3475 (56.5%)	45–75	Prevalence of ideal CVH and CVH metrics
Fang J et al. 2019 [38]	USA	Cross-sectional study	NHANES 2011–2016	6764 (46.5%)	≥20	Prevalence of ideal CVH and CVH metrics
				5278 NHW (49.4)	49.9 (0.5)	
				1486 NHAA (47.6)	44.5 (0.8)	
Folsom AR et al. 2011 [2]	USA	Cross-sectional nested in cohort study	ARIC 1987–1989 (baseline visit)	12744 (43.9%)	45–64; 54.0	Prevalence of ideal CVH and CVH metrics
Foraker RE et al. 2019 [39]	USA	Cross-sectional nested in cohort study	JHS 2000–2004 (baseline examination)	3667 (35.6%)	35–84; 55.1	Prevalence of ideal CVH metrics; EEs of ideal CVH score for income and education
Gao B et al, 2020 [40]	China	Cross-sectional nested in cohort study	National survey 2007–2010	45984 (50.4%)	≥18; 49.2 (15.1)	Prevalence of 5 ideal CVH metrics
Gaye B et al. 2020 [41]	France	Cross-sectional study	2007–2011	68318 (57.7%)	43.3 (13.6)	Prevalence of 6 ideal CVH metrics
Ghimire U et al. 2020 [42]	Nepal	Cross-sectional study	2013	3238 (31.5%)	15–69	Prevalence of ideal CVH and CVH metrics, ideal CVH factors and health behaviors, number of ideal CVH metrics, OR of ideal CVH for education
Gonzalez HM et al. 2016 [43]	USA	Cross-sectional nested in cohort study	HCHS/SOL 2008–2011 (baseline)	15825 (47.8%)	18–74	Prevalence of ideal CVH, CVH metrics and number of ideal CVH metrics
Gonzalez-Rivas JP et al. 2019 [44]	Venezuela	Cross-sectional study	2014–2017	2992 (47%)	≥20; 41.4 (15.8)	Prevalence of ideal CVH metrics and number of ideal CVH metrics
Graciani A et al. 2013 [4]	Spain	Cross-sectional study	ENRICA 2008–2010	11408 (49%)	≥18	Prevalence of ideal CVH metrics, ideal health factors and behaviours, number of ideal CVH metrics, OR of ideal CVH for education
Gupta B et al. 2017 [45]	India	Cross-sectional study	2006–2010	6198 (55.3%)	20–75	Prevalence of ideal CVH and CVH metrics
Harrison S et al. 2019 [46]	Canada	Cross-sectional study	2015–2017	777 (49.8%)	18–65; 41.9 (0.1)	Prevalence of ideal CVH and ideal LS7 metrics and distribution of the LS7 score (0–7)
Isiozor NM et al. 2020 [47]	Finland	Cross-sectional nested in cohort study	KIHD 1984 (baseline)	2577 (100%)	42–60; 53.1 (5.1)	Prevalence of ideal CVH, ideal CVH metrics, ideal health behaviors, and health factors
Jankovic J et al. 2019 [48]	Serbia	Cross-sectional study	NHS 2013	11746 (46%)	≥20; 51.0 (17.4)	Prevalence of ideal CVH metrics, ideal health factors and health behaviors, ORs of ideal CVH for education and Wealth index
Jankovic S et al. 2014 [49]	Republic of Srpska, BH	Cross-sectional study	2010 NHS	4020 (46%)	≥18; 50.2 (17.6)	Prevalence of ideal CVH and CVH metrics, ideal health factors and health behaviors, OR of ideal CVH for education
Kim JI et al. 2013 [50]	USA	Cross-sectional study	HONU project 2009 and 2011	4754 (41.7%)	≥18; 52.1 (16.0)	Prevalence of ideal CVH metrics and number of ideal CVH metrics
Kim JY et al. 2013 [51]	Korea	Cross-sectional nested in cohort study	Seoul Male Cohort Study 1993	12538 (100%)	40–59; 50.0 (5.2)	Prevalence of ideal CVH and CVH metrics
Kulshreshtha A et al. 2013 [52]	USA	Cross-sectional nested in cohort study	REGARDS study 2003–2007 (baseline)	22914 (42.0%)	≥45.0 (65.0)	Prevalence of ideal CVH and CVH metrics
Lawrence EM et al. 2018 [53]	USA	Cross-sectional study	NHANES 2005–2010	689	24–34	Prevalence of ideal CVH and CVH metrics, OR of ideal CVH for education
			Add Health 2007–2008	11200 (51%)		
Liu Y et al. 2014 [54]	China	Cross-sectional nested in cohort study	Kailuan Sudy 2006–2007 (baseline)	95429 (79.7%)	≥18–98; 51.5 (12.5)	Prevalence of ideal CVH and CVH metrics
Lu Y et al. 2015 [8]	China	Cross-sectional study	2013	11996 (64.7%)	≥19; 46.8 (13.0)	Prevalence of ideal CVH and CVH metrics
Machado LBM et al. 2018 [55]	Brasil	Cross-sectional study	2008–2010	13356 (45.3%)	35–74 51.7 (8.9)	Prevalence of ideal CVH and CVH metrics, rPSD of ideal CVH for education and income
Matozinhos FP et al. 2017 [56]	Brasil	Cross-sectional study	2012	41134 (48.4%)	≥18; 41 (0.15)	Prevalence of ideal 6 CVH metrics, PR of ideal CVH for education
Medina-Inojosa JR et al. 2020 [57]	Czech Republic	Cross-sectional nested in cohort study	2014	2074 (47.0%)	25–64; 47.3 (11.3)	Prevalence of ideal CVH and CVH metrics
Moghaddam MM et al. 2014 [58]	Iran	Cross-sectional nested in cohort study	TLGS 2009–2011	4865 (41.2%)	≥20; 41.4 (13.6)	Prevalence of ideal CVH and CVH metrics
Nowicki GJ et al. 2018 [59]	Poland	Cross-sectional study	2015–2016	3901 (41.1%)	35–64; 52.1 (8.2)	Prevalence of ideal CVH and number of ideal CVH metrics
Ogunmoroti O et al. 2017 [60]	USA	Cross-sectional study	BHSF 2014	9056 (26.0%)	43.0 (12.0)	Prevalence of ideal CVH, ideal CVH metrics and number of ideal CVH metrics, OR of ideal CVH for education.
Ommerborn MJ et al. 2016 [61]	USA	Cross-sectional nested in cohort study	JHS 2000–2004 (baseline)	4702	35–84	Prevalence of ideal CVH and numbers of ideal CVH metrics
Patel N et al. 2019 [62]	USA	Cross-sectional study	NHANES 2007–2010	4369 (48.6%)	≥20; 45.0	Prevalence of ideal CVH and number of ideal CVH metrics
Peng Y and Wang Z 2018 [63]	Australia	Cross-sectional study	AHS 2011–2012	7499 (44.4%)	≥18	Prevalence of ideal CVH and ideal health factors and health behaviors
Pilkerton CS et al. 2015 [64]	USA	Cross-sectional nested in cohort study	BRFSS 2011	341659 (47.9)	≥18; 51.1 (0.1)	Prevalence of ideal CVH and CVH metrics
Ren J et al. 2016 [65]	China	Cross-sectional study	SMASH 2011–2015	15350 (50.05%)	18–69; 41.4	Prevalence of ideal CVH and CVH metrics, ORs of ideal CVH for income and education
Seron P et al. 2018 [66]	Latin America^*a*^	Cross-sectional nested in cohort study	CESCAS I 2011–2012	5458 (41,1%)	35–74; 54.8 (10.8)	Prevalence of ideal CVH and CVH metrics
Shay et al. 2012 [67]	USA	Cross-sectional study	NHANES 2003–2008	14515 (50.7%)	≥20	Prevalence of ideal CVH and CVH metrics
Simon M et al. 2017 [9]	France	Cross-sectional study	Paris Prospective Study 3 2008–2012	9012 (61.5%)	50–75; 59.5 (6.3)	Prevalence of ideal CVH and CVH metrics, OR of ideal CVH for education
van Nieuwenhuizen B et al. 2018 [68]	Ghana^*b*^	Cross-sectional study	Multi-centre RODAM study 2012–2015	3510 (38.0%)	18–70; 47.0 (12.0)	Prevalence of ideal CVH and CVH metrics
Velasquez-Melendez G et al. 2015 [69]	Brasil	Cross-sectional study	NHS 2013	34362 (48.7%)	≥18; 43.8 (0.2)	Prevalence of ideal CVH metrics
Wu HY et al. 2013 [70]	China	Cross-sectional study	CHED 2010	1012418 (45.0%)	20–65; 42.7 (6.4)	Prevalence of ideal CVH and CVH metrics
Wu S et al. 2012 [71]	China	Cross-sectional nested in cohort study	Kailuan Study 2006–2007	91698 (79.4%)	18–98; 51.5 (12.4)	Prevalence of ideal CVH and CVH metrics
Zeng Q et al. 2013 [72]	China	Cross-sectional study	DREHM 2009–2012	9962 (55.8%)	20–83; 47.1	Prevalence of ideal CVH and CVH metrics
Zhao Y et al. 2016 [73]	China	Cross-sectional study	2010	2693 (33.4%)	20–80; 51.4 (11.5)	Prevalence of ideal CVH and CVH metrics, ORs of ideal CVH for education and income

Add Health = National Longitudinal Study of Adolescent to Adult Health; AHS = Australian Health Survey; APAC = Asymptomatic Polyvascular Abnormalities Community study; ARIC = Atherosclerosis Risk in Communities; BHSF = Baptist Health South Florida Employee Study; BRFSS = Behavioral Risk Factor Surveillance System; CHED = Chinese Health Examination Database; CHRONICAS = Center of Excellence in Chronic Diseases; CVH = Cardiovascular health; DREHM = Disease Risk Evaluation and Health Management study; ENRICA = Study on Nutrition and Cardio-vascular Risk; Heart SCORE = Heart Strategies Concentrating on Risk Evaluation study; HCHS/SOL = Hispanic Community Health Study/Study of Latinos; HONU = Heart of New Ulm Screening Participants; JHS = Jackson Heart Study; EE = Effect estimate; KIHD = Kuopio Ischemic Heart Disease study; LS7 = Life’s Simple 7; NHAA = non-Hispanic Asian Americans; NHANES = National Health and Nutrition Examination Survey; NHS = National Health Survey; NHW = non-Hispanic white; NOMAS = Northern Manhattan Study; OR = Odds ratio; PR = Prevalence ratio; REGARDS = The Reasons for Geographic And Racial Differences in Stroke; rPSD = Relative predicted score differences; RODAM = Research on Obesity and Diabetes among African Migrants; SMASH = The Shandong province and the Chinese Ministry of Health collaborative Action on Salt reduction and Hypertension; TLGS = Tehran Lipid and Glucose Study; Wealth index = an aggregation of assets and household facilities.

^*a*^Argentina, Chile, and Uruguay

^*b*^Ghanian population in Amsterdam, London and Berlin.

Studies included a total of 2,148,470 participants. Sаmple sizes ranged from 616 to 1,012,418. Two studies [47, 51] included only males. Participants, were adults 18 years and older with an age range from 18 to 107 years. The survey years of the studies included in our review ranged from 1984 to 2017 (33 year period).

### Prevalence of ideal CVH metrics and ideal CVH

**Fig 2:**
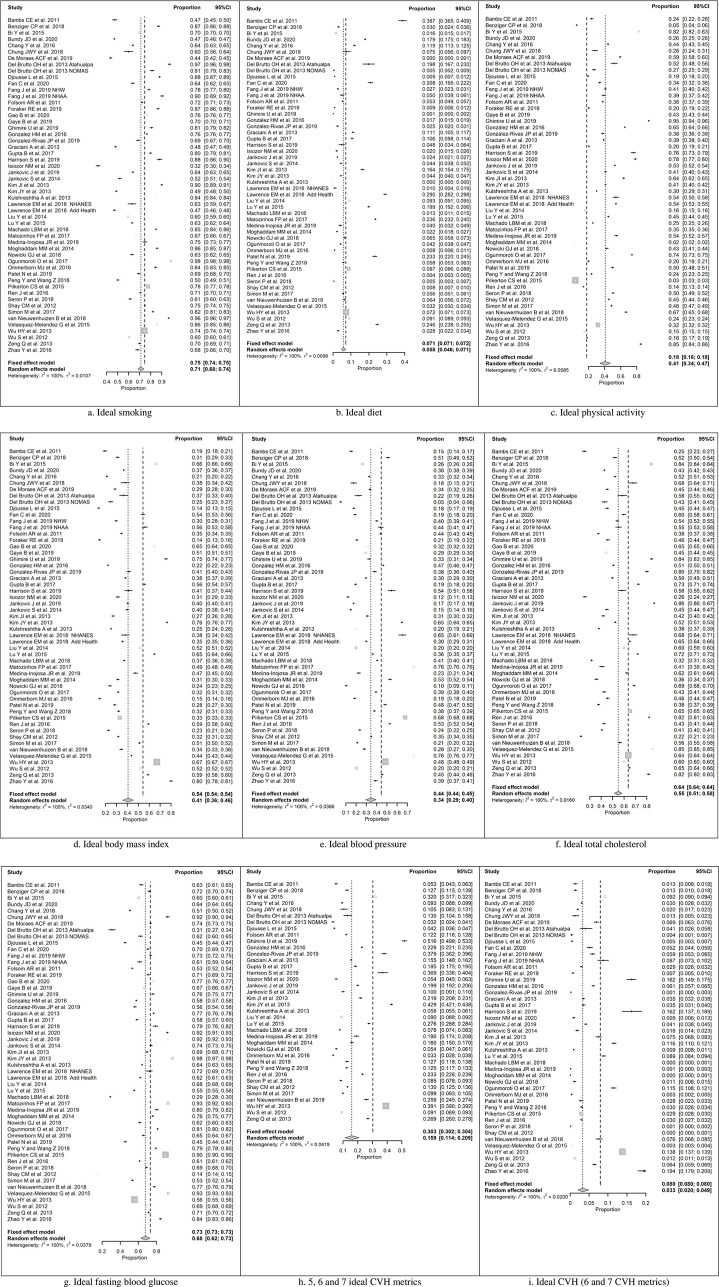
Forest plots showing proportions of ideal cardiovascular health metrics and ideal cardiovascular health. Ideal smoking (A), Ideal diet (B), Ideal physical activity (C), Ideal body mass index (D), Ideal blood pressure (E), Ideal total cholesterol (F), Ideal fasting blood glucose (G), 5, 6 and 7 ideal CVH metrics (H), Ideal CVH (6 and 7 CVH metrics) (I). CVH, Cardiovascular health; CI, confidence interval.

Meta-analyses of each of seven ideal CVH metrics are presented in Fig 2.

The observed proportions for ideal smoking (Fig 2A) ranged from 0.32 to 0.98, and the estimated pooled proportion based on the random-effects model was $\hat{\mu }=0.71$ (95% CI: 0.68 to 0.74). For ideal diet (Fig 2B) the observed double arcsine transformed proportions ranged from 0.003 to 0.569, and the estimated pooled double arcsine transformed proportion based on the random-effects model was $\hat{\mu }=0.234$ (95% CI: 0.194 to 0.274). The back-transformed summary proportion was 0.058 (95% CI: 0.040 to 0.080). The observed proportions for ideal physical activity (Fig 2C) ranged from 0.02 to 0.95, and the estimated pooled proportion based on the random-effects model was $\hat{\mu }=0.41$ (95% CI: 0.34 to 0.47). For ideal body mass index (Fig 2D), the observed proportions ranged from 0.14 to 0.80, and the estimated pooled proportion based on the random-effects model was $\hat{\mu }=0.41$ (95% CI: 0.36 to 0.45). The observed proportions for ideal blood pressure (Fig 2E) ranged from 0.05 to 0.76, and the estimated pooled proportion based on the random-effects model was $\hat{\mu }=0.34$ (95% CI: 0.29 to 0.39). For ideal total cholesterol (Fig 2F), the observed proportions ranged from 0.22 to 0.86, and the estimated pooled proportion based on the random-effects model was $\hat{\mu }=0.55$ (95% CI: 0.51 to 0.59). The observed proportions for ideal fasting blood glucose (Fig 2G) ranged from 0.14 to 0.97, and the estimated pooled proportion based on the random-effects model was $\hat{\mu }=0.68$ (95% CI: 0.62 to 0.73).

In a meta-analysis of 37 studies, for the simultaneous presence of 5, 6, and 7 ideal CVH metrics (Fig 2H), the observed double arcsine transformed proportions ranged from 0.181 to 0.801, and the estimated pooled double arcsine transformed proportion based on the random-effects model was $\hat{\mu }=0.415$ (95% CI: 0.360 to 0.471). The back-transformed summary proportion was 0.159 (95% CI: 0.124 to 0.197).

The prevalence of ideal CVH (defined here as achieving 6 and 7 ideal CVH metrics), intermediate CVH (defined as achieving 2 to 5 ideal CVH metrics), and poor CVH (defined as 0 to 1 ideal CVH metrics) in selected countries are presented in S1 Fig. The prevalence of ideal CVH was low in all observed countries and ranged from 0.5% in the USA Jackson Heart Study [36] to 15% in the Chinese Health Examination Database study [70]. As many as 15 out of the 21 studies conducted worldwide reported less than 5% prevalence of ideal CVH. Most participants had intermediate CVH with the prevalence ranging from 70% in the USA Heart SCORE study [29] to 93% in the Serbian Health Survey [48]. The prevalence of poor CVH ranged from 1% in the Korean Seoul Male Cohort Study [51] to 29% in the USA Heart SCORE study [29].

The prevalence of ideal health behaviors and the prevalence of ideal health factors from 10 studies with available data are presented in S2 Fig. The percentage of subjects who achieved the 4 ideal behaviors or lifestyles (smoking, diet, physical activity, and BMI) was lower than the percentage of those with the 4 ideal health or biological factors (blood pressure, total cholesterol, fasting blood glucose, and smoking) in all observed studies, except one [29]. As defined by the AHA, given the importance of abstinence from smoking and smoking cessation to health promotion, this metric appears in both health behaviors and health factors [1].

For the ideal CVH (6 and 7 CVH metrics) a total of 44 studies were included in the meta-analysis (Fig 2I), and the observed double arcsine transformed proportions ranged from 0.015 to 0.415. The estimated pooled double arcsine transformed proportion based on the random-effects model was $\hat{\mu }=0.188$ (95% CI: 0.154 to 0.220). The back-transformed summary proportion was 0.033 (95% CI: 0.022 to 0.046).

For all ideal CVH metrics, as well as for the presence of 5 and more ideal CVH metrics and ideal CVH, the true outcomes (proportions) appear to be heterogeneous indicating the need for moderator (subgroup) analysis.

### Prevalence of ideal CVH metrics and ideal CVH according to gender and age

Where data was available, participants were stratified by gender (female and male) and moderator analysis was performed for each of 7 ideal CVH metrics, for the presence of 5 and more ideal CVH metrics, and for ideal CVH (6 and 7 ideal CVH metrics) (Table 2). Gender was a statistically significant moderator of ideal smoking (proportion of 0.81 in females and 0.60 in males; QM = 22.12, p < 0.001), and ideal blood pressure (proportion of 0.42 in females and 0.30 in males; QM = 8.94, p = 0.003). Gender was also a statistically significant moderator of ideal CVH with the proportion of 0.06 in females and 0.03 in males (QM = 4.61, p = 0.032), and for the simultaneous presence of 5, 6 and 7 CVH metrics with the proportion of 0.21 in females and 0.13 in males (QM = 6.44, p = 0.011).

**Table 2:** Moderator (subgroup) analyses of ideal cardiovascular health metrics by gender.

Ideal CVH metric	Gender	No. of studies		Heterogeneity			Meta-analysis			Test of moderators	
			Population	P-value^*a*^	I^2^	Model	Proportion	95% CI	P-value	QM	P-value^*b*^
Smoking	F	33	710,944	<0.001	99.99%	Random	0.814	0.746, 0.881	<0.001	22.121	<0.001
	M	35	829,878	<0.001	99.94%	Random	0.601	0.543, 0.658	<0.001		
Diet	F	32	684,598	<0.001	99.95%	Random	0.069	0.043, 0.101	<0.001	1.131	0.288
	M	34	811,803	<0.001	99.94%	Random	0.050	0.031, 0.074	<0.001		
Physical activity	F	32	684,598	<0.001	99.98%	Random	0.382	0.294, 0.470	<0.001	1.319	0.251
	M	34	811,803	<0.001	99.97%	Random	0.451	0.374, 0.528	<0.001		
Body mass index	F	33	710,944	<0.001	99.95%	Random	0.465	0.401, 0.530	<0.001	1.129	0.288
	M	34	827,301	<0.001	99.95%	Random	0.417	0.357, 0.478	<0.001		
Blood pressure	F	33	710,944	<0.001	99.93%	Random	0.420	0.365, 0.474	<0.001	8.944	0.003
	M	35	829,878	<0.001	99.96%	Random	0.297	0.239, 0.356	<0.001		
Total cholesterol	F	32	689,719	<0.001	99.96%	Random	0.569	0.502, 0.636	<0.001	0.054	0.817
	M	34	809,969	<0.001	99.95%	Random	0.580	0.519, 0.640	<0.001		
Fasting blood glucose	F	33	710,944	<0.001	99.92%	Random	0.733	0.690, 0.776	<0.001	1.910	0.167
	M	35	829,878	<0.001	99.96%	Random	0.684	0.629, 0.739	<0.001		
5, 6, and 7 metrics	F	27	648,071	<0.001	99.95%	Random	0.213	0.159, 0.272	<0.001	6.442	0.011
	M	29	837,000	<0.001	99.93%	Random	0.127	0.092, 0.166	<0.001		
Ideal CVH (6 and 7)	F	21	620,728	<0.001	99.95%	Random	0.055	0.032, 0.083	<0.001	4.607	0.032
	M	23	754,906	<0.001	99.83%	Random	0.026	0.015, 0.039	<0.001		

CVH = Cardiovascular health; F = Female; M = Male

^*a*^Test for heterogeneity (Q)

^*b*^Test of moderators (QM)

**Fig 3:**
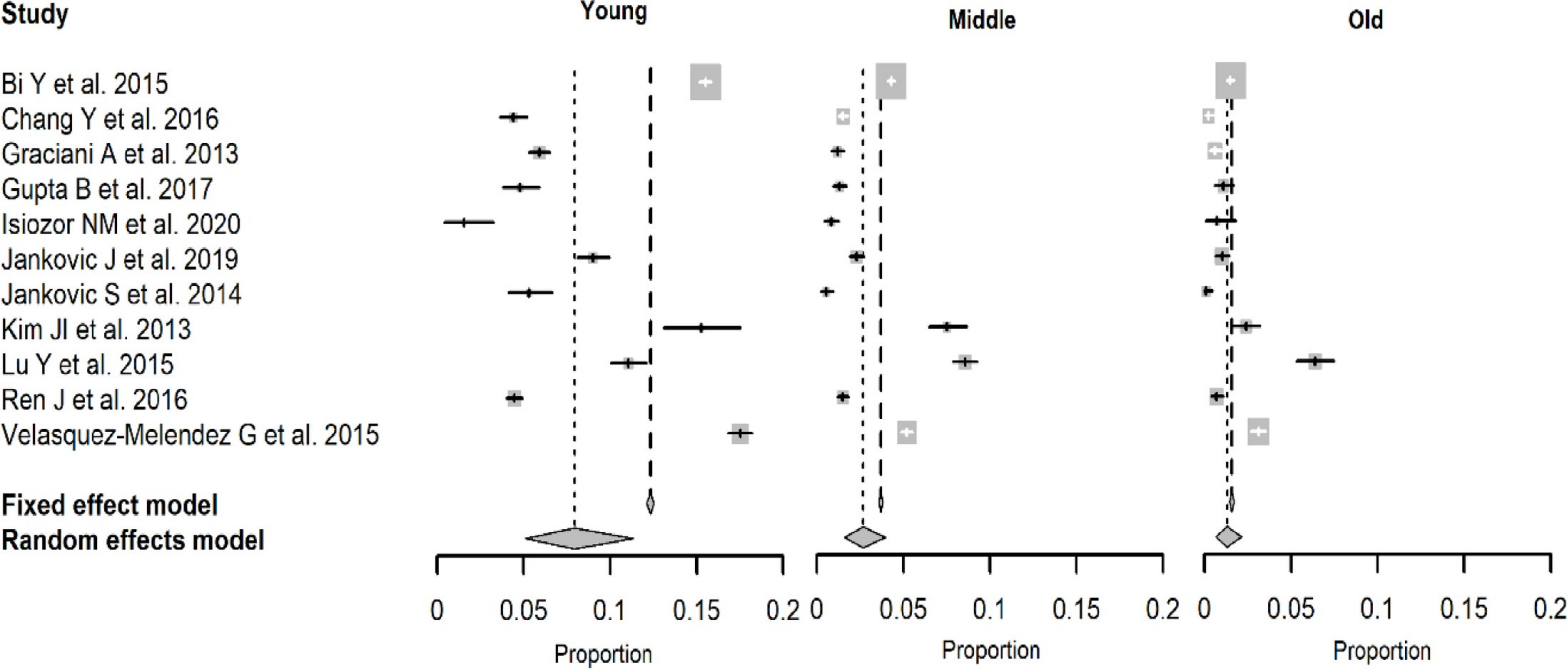
Forest plots depicting proportions with their confidence intervals of ideal cardiovascular health by age group.

A total of 11 studies had available data and were included in the analysis of age and ideal CVH (6 and 7 ideal CVH metrics) (Fig 3). The back-transformed summary proportions were: 0.08 (95% CI: 0.05 to 0.11) for the young age group, 0.03 (95% CI: 0.01 to 0.04) for the middle age group, and 0.01 (95% CI: 0.01 to 0.02) for the older age group. Test of moderators revealed that the difference between the three subgroups is significant (QM = 24.24, DF = 2, p < 0.001). The better CVH was seen in younger and middle-aged adults compared to older participants.

### Ideal CVH according to socioeconomic characteristics

The results of 14 studies describing the association between socioeconomic variables (education, income, and Wealth index), and the prevalence of ideal CVH are presented in Table 3.

**Table 3:** Studies describing the association between socioeconomic variables and prevalence of ideal cardiovascular health.

First author, year	Outcome	Adjustment for potential confounding	Measure of socioeconomic status (95% CI)
Benziger CP et al. 2018 [30]	Ideal CVH metrics (5–7)	Age, sex, site	Education PR^*a*^ 0.96 (0.68, 1.35)
			Wealth index PR^*a*^ 0.75 (0.55, 1.01)
			Wealth index PR^*b*^ 0.73 (0.56, 0.95)
Chang Y et al. 2016 [33]	Ideal CVH metrics (5–7 vs. 0–4)	Age, sex, marital status	Education OR^*a*^ 1.95 (1.70, 2.25)
			Family income OR^*a*^ 1.70 (1.30, 2.20)
Foraker RE et al. 2019 [39]	CVH score: sum of 7 ideal CVH metrics ranging from 0 (worst) to 14 (best) points	Age, sex, neighborhood income or education where appropriate	Individual income EE^*a*^^*c*^ 0.31 (0.24, 0.37)
			Neighborhood income EE^*a*^^*c*^ 0.19 (0.09, 0.28)
			Education (bachelor) EE^*a*^^*c*^ 0.67 (0.43, 0.91)
			Education (graduate) EE^*a*^^*c*^ 0.93 (0.69, 1.16)
Ghimire U et al. 2019 [42]	Ideal CVH metrics (5–7)	Age, sex, marital status, residence, ethnicity	Education OR^*a*^ 0.74 (0.44, 1.25)
Graciani A et al. 2013 [4]	Ideal CVH metrics (>6 vs. <1)	Age, sex, self-rated health, and use of health care system	Education OR^*a*^ 2.60 (1.45, 4.64)
Jankovic J et al. 2019 [48]	Ideal CVH metrics (6–7 vs. 0–5)	Age, sex, type of settlement, marital status	Education OR^*a*^ 3.57 (2.36, 5.40)
			Wealth index OR^*a*^ 1.43 (1.08, 1.88)
			Wealth index OR^*b*^ 1.22 (0.91, 1.63)
Jankovic S et al. 2014 [49]	Ideal CVH metrics (5–7 vs. 0–4)	Age, sex, type of settlement, marital status, employment	Education OR^*a*^ 2.3 (1.5, 3.5)
Lawrence EM et al. 2018 [53]	CVH (ideal vs.poor)	Age, sex	Education OR^*a*^ 4.98 (3.41, 7.28)
Machado LBM et al. 2018 [55]	CVH score: sum of 7 ideal CVH metrics ranging from 0 to 7	Age, sex, race, educational, family income and study site effect	Education rPSD^*d*^ −17.2 (−20.0, −14.2)
		Age, sex, race, educational level, and study site effect	Family income^*d*^ rPSD − 4.4 (−7.2, −1.6)
Matozinhos FP et al. 2017 [56]	Ideal CVH metrics (5–6)		Education women PR^*d*^ 0.28 (0.23, 0.33)
			Education men PR^*d*^ 0.27 (0.21, 0.33)
Ogunmoroti O et al. 2017 [60]	Ideal CVH metrics (6–7)	Age, sex, ethnicity	Education OR^*a*^ 0.29 (0.19, 0.44)
Ren J et al. 2016 [65]	Ideal CVH metrics (≥5 vs. ≤4)	Age, sex	Personal income + education OR^*a*^ 1.85 (1.40,2.45)
Simon M et al. 2017 [9]	CVH (ideal vs. poor)		Education OR^*a*^^*e*^ 5.20 (4.07, 6.77)
Zhao Y et al. 2016 [73]	Ideal CVH (7 ideal CVH metrics)	History of hypertension, diabetes, dyslipidemia, and CVD	Education OR^*f*^ 1.33 (0.64, 2.74)
			Family income OR^*f*^ 0.93 (0.59, 1.48)

CI = Confidence intervals; CVH = Cardiovascular health; EE = Effect estimate; PR = Prevalence ratio; rPSD = Relative predicted score differences; OR = Odds ratio; Wealth index = an aggregation of assets and household facilities.

^*a*^highest compared to lowest

^*b*^middle compared to lowest

^*c*^per category increase

^*d*^lowest compared to highest

^*e*^women vs. men

^*f*^highest vs. moderate.

Results from most studies showed that participants with the highest education had a greater number of ideal CVH metrics, i.e. better CVH in comparison with those with the lowest education [4, 9, 33, 39, 48, 49, 53, 56, 73]. In a study by Foraker et al. [39] individuals with a bachelor’s degree, had on average, a CVH score of 0.67 points higher compared with those with high school or less education. Similarly, persons with graduate/professional degrees had on average a CVH score of 0.93 points higher than their less-than-high educated school counterparts. Machado et al. [55] found that individuals with less than high school education had lower ideal CVH scores than college-educated individuals. In a study by Matozinhos et al. [56] lower levels of education were associated with a lower prevalence of ideal CVH, regardless of gender, compared to those with a higher level of education. In contrast, Ongunmoroti et al. [60] found that lower education was associated with better CVH, while Benziger et al. [30] and Zhao et al. [73] did not find any statistically significant association between education and ideal CVH (Table 3).

Only a few studies assessed the relationship between income and CVH. Foraker et al. [39] found that those with higher individual and neighborhood levels of income had higher CVH scores, i.e. better CVH. There was an average increase in CVH score of 0.31, and 0.19 points associated with each 1-category increase in individual income, and neighborhood income, respectively. Better results, considering the number of ideal CVH metrics were also achieved in persons with more family income living in Hong Kong [33]. Machado et al. [55] demonstrated that low (<1245 USD) family income was associated with lower ideal CVH scores compared to those with high (≥3320 USD) family income. In contrast, in a rural area of Northwest China, no associations were found between family income and ideal CVH [73] (Table 3).

In one study conducted in China, higher socioeconomic status (defined according to both personal income and years of education) was associated with an increasing prevalence of meeting 5 or more ideal CVH metrics in women but not in men [65]. Only two studies included in our review assessed the association between Wealth index (an aggregation of assets and household facilities) and ideal CVH. While Jankovic et al. [48] noted a statistically positive association between Wealth index and ideal CVH, Benziger et al. [30] found the opposite (Table 3).

### Quality assessment

According to both applied quality assessment tools (NIH-QAT and tool specifically designed for prevalence studies), almost all included studies were regarded as having a low risk of bias (S3 and S4 Tables). Agreement on the quality assessment between the two reviewers was high.

## Discussion

Our study is the second meta-analysis ever done that calculated the pooled prevalence estimates of the seven ideal CVH metrics and overall ideal CVH according to the AHA’s guideline, and the first systematic review that examined the association between socioeconomic status (SES) and ideal CVH. Since a considerable number of studies on the prevalence of ideal CVH have been published in the last few years our study provides up-to-date information that could be useful for policymakers, clinicians, researchers, communities, and other stakeholders to understand and implement the most effective approaches to improve CVH in populations.

This study showed a low prevalence of ideal CVH defined as 6 and 7 ideal metrics (3.3%) that is in line with a previous systematic review [15]. When a less strict definition of ideal CVH was applied (5 to 7 ideal metrics) about 16% of our participants had an overall ideal CVH like in recent meta-analysis [19]. In most countries poor CVH (0–1 ideal metrics) was more frequent than ideal CVH (6 and 7 ideal metrics), while intermediate CVH (2–5 ideal metrics) was the most prevalent. Except in one study [29], we also observed a lower prevalence of ideal health behaviors (ranged from 0.1% to 2%) compared with the prevalence of ideal health factors (ranged from 1.4% to 16.4%).

Among seven ideal CVH metrics, smoking was the best metric (71%), followed by fasting blood glucose (68%), total cholesterol (55%), physical activity (41%), BMI (41%), and blood pressure (34%), while the poorest CVH metric was healthy diet (5.8%).

The results of our meta-analyses are summary proportions of ideal CVH and ideal CVH metrics from studies conducted during different study periods, ranging from 1984 to 2017. We are aware that the CVH status has changed during the observed period and several cohort studies assessing trends in ideal CVH and CVH metrics documented that finding. Patel et al. [62] examined the trends in ideal CVH during economic recession (2007–2010) and subsequent economic recovery (2011–2016) among American adults and noted a decline in ideal CVH score that was primarily driven by the increased prevalence of obesity and poor fasting glucose. A Danish study [5], using data from six cross-sectional studies conducted in an adult population aged 30–64 years in Denmark from 1978 to 2006, reported an increasing trend in ideal CVH. Huffman et al. [74] analyzed CVH behavior and health factor changes from 1988 to 2008 and showed modest, further declines in tobacco consumption, high cholesterol, and high blood pressure, offset by increases in obesity and dysglycemia. The high proportions of people with favorable smoking status in this study (71%) could be a reflection of comprehensive tobacco control policies and a significant decrease of smoking worldwide [74]. The prevalence of ideal fasting blood glucose metric (68%) like in reviews by Younus et al. [15] and Peng et al. [19] was also high but trend results suggest its decline [62, 74]. This high optimal prevalence of fasting blood glucose may partly be explained by the slight improvement of physical activity and dietary pattern [74–76], which were found to be risk factors for elevated fasting blood glucose and diabetes [77]. In our review, 55% of participants had ideal total cholesterol that is in line with the recent meta-analysis [19]. Total cholesterol levels were decreasing, partly due to the increasing use of lipid-lowering drugs [78, 79]. We reported that 41% of participants had ideal blood pressure that is consistent with findings from the recent US nationally representative survey (42.3%) [80], but higher in comparison with results of a recent meta-analysis (34.6%) [19] and the China national hypertension survey (35.5%) [81]. Our results of ideal physical activity (41%), and ideal BMI (41%) are also in accordance with a previous meta-analysis by Peng et al. [19]. In our study, the poorest CVH metric was a healthy diet (5.8%) like in both previous reviews [15, 19] and almost all individual studies that examined ideal CVH. Although the prevalence of a healthy diet is extremely low, the question of its relevance to ideal CVH does not arise. A healthy diet is one of 7 CVH metrics proposed by AHA, essential for keeping people healthy across the lifespan which could be successfully improved [1]. Unfortunately, physical activity levels and low diet quality scores changed minimally during the observed time [74]. Hence, successful prevention efforts for improvement of healthy diet and physical activity are needed. This should result in improvement in BMI, blood pressure, fasting blood glucose, and overall CVH status and reducing the related CVD burden.

In this study, women had twice higher ideal CVH (6%) than men (3%) and a higher percentage of 5 and more ideal CVH metrics (21% in women vs. 13% in men) that is in line with findings from a recent meta-analysis [19]. Several studies have specifically investigated gender disparities in the distribution of ideal CVH and discussed the potential reasons behind that [7, 9, 31]. There is evidence that women attended primary care (general practitioner/family physician) and preventive services more often than men [82–84]. Simon et al. [9] hypothesized that more frequent contact of women with the health care system throughout their life (e.g. concerning contraception, pregnancy, and child care) is an opportunity to be more sensitive to health promotion and prevention, contributing to a greater willingness to follow public health and medical recommendations. Subgroup analysis also revealed that gender was a statistically significant moderator of ideal smoking, with a higher percentage in females (81%) compared to males (60%). It could partly be explained by less frequent smoking in women than men. However, epidemiological evidence suggests that smoking is a stronger cardiovascular risk factor in women [85, 86]. This finding and increasing trend in female smoking point out that tobacco control policies should include items specifically targeted at women. Also, there was a statistically significant difference in ideal blood pressure between females (42%) and males (30%) which is in concert with a higher prevalence of raised blood pressure in men compared to women worldwide, but this difference was only statistically significant in the Region of the Americas and the European Region [87].

From the beginning of this century, considerable efforts have been made to improve understanding of the sex/gender differences in CVD and to heighten awareness of heart disease in women [7]. Policy and prevention efforts, to be successful, need to be investigated and targeted within genders. A better understanding of sex differences in CVD is needed to prevent and treat CVD more efficiently in both gender. Continued efforts are required to unravel the belief that CVD is a man’s disease [88].

Like in themeta-analysis by Peng et al. [19] our results showed that younger adults had higher overall CVH than older counterparts. We found statistically significant differences in the summary prevalence of ideal CVH among younger (8%), middle-aged (3%), and older participants (1%). The highest prevalence of ideal CVH observed in younger subjects could be explained mostly by their better ideal health factors, although this is in contrast to the poor results on health behaviors reported elsewhere [4, 89]. Comparing to other two groups, the oldest participants had lower levels of almost all ideal CVH metrics except for smoking and diet [4, 49]. Nevertheless, the young people should be a priority objective for primordial prevention that may prevent worsening of their current CVH in the future.

Although literature data highlights the relevance of SES as determinants of health in both high- income and low- and middle-income countries [90–92], only a few studies have examined the association between ideal CVH and SES. Accumulating evidence supports the notion that social environment (SES and social networks) shape the personal lifestyle choices such as physical activity, nutritional choices, and smoking which profoundly influence CVH risk factors. However, the cumulative impact of the environment on CVD risk has been difficult to assess and the mechanisms by which some environment factors influence CVD remain obscure [93]. Education, income or aggregation of assets and household facilities known as the Wealth index [94, 95] are most commonly used as proxies for SES. Results from many studies included in our systematic review showed that participants with higher education had a greater number of ideal CVH metrics, i.e. better CVH in comparison with those with lower education. Olsen et al. [5] reported an increasing trend in ideal CVH with a more unfavorable risk profile among persons with low educational levels. Another study [96] found that additional years of education are associated with better CVH. The Tromso study [97] demonstrated the most unfavorable CVD risk factors in the lowest educational group. Our results are also in accordance with the results from the world’s largest population-based cross-sectional study performed in the 50 states of the USA [3]. Only two studies [30, 48] included in our review assessed the association between Wealth index and ideal CVH. Jankovic et al. [48] found a positive association, while the opposite results were noted in the study by Benziger et al. [30]. Several previous studies failed to find any significant association between the Wealth index and CVH score [98, 99]. Overall, the results of this review suggest that those with higher levels of SES have better CVH and a possible explanation could be the fact that people with higher education or income are more prone to afford healthier foods and exercise more compared with those with lower education or income. Also, better-off people compared with those worse-off are more likely to afford high-quality health care.

The strength of our study is that we calculated the pooled prevalence estimates for the 7 ideal CVH metrics and overall CVH according to age and sex on a large sample size (over 2 million people). Besides, this is the first systematic review on the relationship between participants’ SES and ideal CVH. However, several limitations should be briefly stressed. First, the results of meta-analyses of ideal CVH metrics and ideal CVH showed a great heterogeneity across studies and it could be explained by the variance in the measurements of ideal CVH metrics in assessed studies, differences in the study populations in terms of gender, age, SES, geographic distribution, lifestyle patterns, and a difference in survey years for each of the included studies. Second, some CVH metrics (smoking, dietary intake, and physical activity) were self-reported measures, which carried an inherent degree of bias. Third, not all studies followed the metric definitions of AHA, especially for a healthy diet or physical activity which may preclude comparisons between studies. To address heterogeneity at least a part, we performed subgroup analyses of each of the 7 ideal CVH metrics and their clustering (6 and 7; and 5 and more ideal metrics) by gender and age groups.

### Conclusion

Our systematic review and meta-analysis provided evidence that ideal CVH is low worldwide. We found that gender was a statistically significant moderator of ideal CVH, cluster of 5 and more ideal CVH metrics, ideal smoking, and ideal blood pressure (the better values were observed in females). Also, younger, more educated and better-off individuals had a greater number of ideal CVH metrics, i.e. better ideal CVH in comparison with middle or old age participants, less educated and worse-off. To achieve the improvement of the CVH metrics and the overall ideal CVH, nationwide primordial prevention efforts at the population and individual levels are urgently needed and should focus on the diet, as well as those components which showed gender inequalities, such as smoking and blood pressure. Targeting public health interventions to improve CVH status among less educated and more disadvantaged individuals may have substantial societal implications.

## Supporting information

S1 Fig Distribution of the cardiovascular health status (ideal, intermediate, and poor) in different countries. (DOCX)

S2 Fig Ideal health behaviors and ideal health factors in observed studies. (DOCX)

S1 Table Definition of the AHA 2020 cardiovascular health metrics. (DOCX)

S2 Table PRISMA checklist. (DOCX)

S3 Table Quality assessment of the included studies using the NIH QAT for observational cohort and cross-sectional studies. (DOCX)

S4 Table Quality assessment of the included studies using the risk of bias tool for prevalence studies. (DOCX)
