# Supplementary material for: Demographic and socioeconomic inequalities in ideal cardiovascular health: A systematic review and meta-analysis
Source: PLoS One. 2021 Aug 11;16(8):e0255959. doi: 10.1371/journal.pone.0255959 (PMC8357101; doi:10.1371/journal.pone.0255959)
Supplement: S1 Fig — (DOCX) [file pone.0255959.s001.docx]

S1 Fig. Distribution of the cardiovascular health status (ideal, intermediate, and poor) in different countries

AHS = Australian Health Survey; ARIC = Atherosclerosis Risk in Communities; CHED = Chinese Health Examination Database; CHRONICAS = Center of Excellence in Chronic Diseases; CVH = Cardiovascular health; ENRICA = Study on Nutrition and Cardio-vascular Risk; Heart SCORE = Heart Strategies Concentrating on Risk Evaluation; HONU = Heart of New Ulm Screening Participants; JHS = Jackson Heart Study; NHANES = National Health and Nutrition Examination Survey; NHS = National Health Survey; RODAM = Research on Obesity and Diabetes among African Migrants; REGARDS = The Reasons for Geographic And Racial Differences in StrokeSMCS = Seoul Male Cohort StudyTLGS = Tehran Lipid and Glucose Study.
