# Supplementary material for: Demographic and socioeconomic inequalities in ideal cardiovascular health: A systematic review and meta-analysis
Source: PLoS One. 2021 Aug 11;16(8):e0255959. doi: 10.1371/journal.pone.0255959 (PMC8357101; doi:10.1371/journal.pone.0255959)
Supplement: S2 Fig — (DOCX) [file pone.0255959.s002.docx]

S2 Fig. Ideal health behaviors and ideal health factors in observed studies
