# Supplementary material for: Demographic and socioeconomic inequalities in ideal cardiovascular health: A systematic review and meta-analysis
Source: PLoS One. 2021 Aug 11;16(8):e0255959. doi: 10.1371/journal.pone.0255959 (PMC8357101; doi:10.1371/journal.pone.0255959)
Supplement: S1 Table — (DOCX) [file pone.0255959.s003.docx]

S1 Table. Definition of the AHA 2020 Cardiovascular Health Metrics

| CVH metric | Ideal | Intermediate | Poor |
| --- | --- | --- | --- |
| Smoking | Never or former >1 year | Former ≤1 year | Current |
| Healthy diet[*^a^*](http://jaha.ahajournals.org/content/3/3/e000635/T1.expansion.html#fn-2) | 4 to 5 components | 2 to 3 components | 0 to 1 components |
| Physical activity | ≥150 min/week moderate, or ≥75 min/week vigorous, or ≥150 min/week moderate + vigorous | 1–149 min/week moderate, or 1–74 min/week vigorous, or 1–149 min/week moderate + vigorous | None |
| Body mass index | <25 kg/m^2^ | 25 to 29.9 kg/m^2^ | ≥30 kg/m^2^ |
| Blood pressure | <120/<80 mm Hg untreated | SBP 120 to 139 or DBP 80 to 89 mm Hg or treated to ideal level | SBP ≥140 or DBP ≥90 mm Hg |
| Total cholesterol | <200 mg/dL untreated | 200 to 239 mg/dL or treated to ideal level | ≥240 mg/dL |
| Fasting glucose | <5.55 mmol/L (<100 mg/dL) untreated | 5.55 to 6.94 mmol/L (100 to 125 mg/dL) or treated to ideal level | ≥6.99 mmol/L (≥126 mg/dL) |

AHA = American Heart Association; CVH = Cardiovascular health; SBP = systolic blood pressure; DBP = diastolic blood pressure.

[*^a^*](http://jaha.ahajournals.org/content/3/3/e000635/T1.expansion.html#fn-2)Five components defined as consumption of fruits and vegetables (≥4.5 cups/day), whole grain (≥3 one ounce servings/day), sodium (<1500 mg/day), sugar-sweetened beverages (≤36 oz/week), and fish (≥2 3.5 oz servings/week). 1 oz represents approximately 28.4 gr.
