# Supplementary material for: Demographic and socioeconomic inequalities in ideal cardiovascular health: A systematic review and meta-analysis
Source: PLoS One. 2021 Aug 11;16(8):e0255959. doi: 10.1371/journal.pone.0255959 (PMC8357101; doi:10.1371/journal.pone.0255959)
Supplement: S3 Table — (DOCX) [file pone.0255959.s005.docx]

S3 Table. Quality assessment of the included studies using the NIH-QAT

| First author, year, reference | Item 1 | Item 2 | Item 3 | Item 4 | Item 5 | Item 6 | Item 7 | Item 8 | Item 9 | Item 10 | Item 11 | Item 12 | Item 13 | Item 14 | Score | Quality |
| --- | --- | --- | --- | --- | --- | --- | --- | --- | --- | --- | --- | --- | --- | --- | --- | --- |
| Bambs 2011 [29] | ● | ● | ● | ● | ○ | NA | NA | NA | ● | NA | ● | NA | NA | ● | 0.87 | Good |
| Benziger 2018 [30] | ● | ● | ● | ● | ○ | NA | NA | NA | ● | NA | ● | NA | NA | ● | 0.87 | Good |
| Bi 2015 [31] | ● | ● | ● | ● | ○ | NA | NA | NA | ● | NA | ● | NA | NA | ○ | 0.75 | Good |
| Bundy 2020 [32] * | ● | ● | ● | ○ | ○ | NA | NA | NA | ● | NA | ● | NA | NA | ○ | 0.62 | Fair |
| Chang 2016 [33] | ● | ● | ● | ● | ○ | NA | NA | NA | ● | NA | ● | NA | NA | ● | 0.87 | Good |
| Chung 2018 [34] | ● | ● | ● | ● | ● | NA | NA | NA | ● | NA | ● | NA | NA | ● | 1.00 | Good |
| De Moraes 2019 [10] | ● | ● | ● | ● | ○ | NA | NA | NA | ● | NA | ● | NA | NA | ● | 0.87 | Good |
| Del Brutto 2013 [35] | ● | ● | ● | ● | ○ | NA | NA | NA | ● | NA | ● | NA | NA | ● | 0.87 | Good |
| Djousse 2015 [36]* | ● | ● | ● | ● | ○ | NA | NA | NA | ● | NA | ● | NA | NA | ○ | 0.75 | Good |
| Fan 2020 [37]* | ● | ● | ● | ● | ○ | NA | NA | NA | ● | NA | ● | NA | NA | ○ | 0.75 | Good |
| Fang 2019 [38] | ● | ● | ● | ● | ○ | NA | NA | NA | ● | NA | ● | NA | NA | ● | 0.87 | Good |
| Folsom 2011 [2]* | ● | ● | ● | ● | ○ | NA | NA | NA | ● | NA | ● | NA | NA | ○ | 0.75 | Good |
| Foraker 2019 [39]* | ● | ● | ● | ● | ● | NA | NA | NA | ● | NA | ● | NA | NA | ● | 1.00 | Good |
| Gao 2020 [40]* | ● | ● | ● | ● | ● | NA | NA | NA | ● | NA | ● | NA | NA | ○ | 0.87 | Good |
| Gaye 2020 [41] | ● | ● | ● | ● | ○ | NA | NA | NA | ● | NA | ● | NA | NA | ● | 0.87 | Good |
| Ghimire 2020 [42] | ● | ● | ● | ● | ○ | NA | NA | NA | ● | NA | ● | NA | NA | ● | 0.87 | Good |
| Gonzalez 2016 [43]* | ● | ● | ● | ● | ○ | NA | NA | NA | ● | NA | ● | NA | NA | ● | 0.87 | Good |
| Gonzalez-Rivas 2019 [44] | ● | ● | ● | ● | ○ | NA | NA | NA | ● | NA | ● | NA | NA | ● | 0.87 | Good |
| Graciani 2013 [4] | ● | ● | ● | ● | ○ | NA | NA | NA | ● | NA | ● | NA | NA | ● | 0.87 | Good |
| Gupta 2017 [45] | ● | ● | ● | ● | ○ | NA | NA | NA | ● | NA | ● | NA | NA | ● | 0.87 | Good |
| Harrison 2019 [46] | ● | ● | ● | ● | ○ | NA | NA | NA | ● | NA | ● | NA | NA | ● | 0.87 | Good |
| Isiozor 2020 [47]* | ● | ● | ● | ● | ○ | NA | NA | NA | ● | NA | ● | NA | NA | ○ | 0.75 | Good |
| Jankovic 2019 [48] | ● | ● | ● | ● | ● | NA | NA | NA | ● | NA | ● | NA | NA | ● | 1.00 | Good |
| Jankovic 2014 [49] | ● | ● | ● | ● | ● | NA | NA | NA | ● | NA | ● | NA | NA | ○ | 0.87 | Good |
| Kim 2013 [50] | ● | ● | ● | ● | ○ | NA | NA | NA | ● | NA | ● | NA | NA | ○ | 0.75 | Good |
| Kim 2013 [51]* | ● | ● | ● | ● | ○ | NA | NA | NA | ● | NA | ● | NA | NA | ○ | 0.75 | Good |
| Kulshreshtha 2013 [52]* | ● | ● | ● | ● | ○ | NA | NA | NA | ● | NA | ● | NA | NA | ○ | 0.75 | Good |
| Lawrence 2018 [53] | ● | ● | ● | ● | ○ | NA | NA | NA | ● | NA | ● | NA | NA | ● | 0.87 | Good |
| Liu 2014 [54]* | ● | ● | ● | ● | ○ | NA | NA | NA | ● | NA | ● | NA | NA | ○ | 0.75 | Good |
| Lu 2015 [8] | ● | ● | ● | ● | ○ | NA | NA | NA | ● | NA | ● | NA | NA | ○ | 0.75 | Good |
| Machado 2018 [55] | ● | ● | ● | ● | ○ | NA | NA | NA | ● | NA | ● | NA | NA | ● | 0.87 | Good |
| Matozinhos 2017 [56] | ● | ● | ● | ● | ○ | NA | NA | NA | ● | NA | ● | NA | NA | ○ | 0.75 | Good |
| Medina-Inojosa 2020 [57]* | ● | ● | ● | ● | ○ | NA | NA | NA | ● | NA | ● | NA | NA | ○ | 0.75 | Good |
| Moghaddam 2014 [58]* | ● | ● | ○ | ● | ○ | NA | NA | NA | ● | NA | ● | NA | NA | ○ | 0.62 | Fair |
| Nowicki 2018 [59] | ● | ● | ● | ● | ○ | NA | NA | NA | ● | NA | ● | NA | NA | ● | 0.87 | Good |
| Ogunmoroti 2017 [60] | ● | ● | ● | ● | ○ | NA | NA | NA | ● | NA | ● | NA | NA | ● | 0.87 | Good |
| Ommerborn 2016 [61]* | ● | ● | ● | ● | ○ | NA | NA | NA | ● | NA | ● | NA | NA | ○ | 0.75 | Good |
| Patel 2019 [62] | ● | ● | ● | ● | ○ | NA | NA | NA | ● | NA | ● | NA | NA | ○ | 0.75 | Good |
| Peng 2018 [63] | ● | ● | ● | ● | ○ | NA | NA | NA | ● | NA | ● | NA | NA | ○ | 0.75 | Good |
| Pilkerton 2015 [64]* | ● | ● | ● | ● | ● | NA | NA | NA | ● | NA | ● | NA | NA | ● | 1.00 | Good |
| Ren 2016 [65] | ● | ● | ● | ● | ● | NA | NA | NA | ● | NA | ● | NA | NA | ● | 1.00 | Good |
| Seron 2018 [66]* | ● | ● | ● | ● | ● | NA | NA | NA | ● | NA | ● | NA | NA | ○ | 0.87 | Good |
| Shay 2012 [67] | ● | ● | ● | ● | ● | NA | NA | NA | ● | NA | ● | NA | NA | ○ | 0.87 | Good |
| Simon 2017 [9] | ● | ● | ● | ● | ○ | NA | NA | NA | ● | NA | ● | NA | NA | ● | 0.87 | Good |
| van Nieuwenhuizen 2018 [68] | ● | ● | ● | ● | ○ | NA | NA | NA | ● | NA | ● | NA | NA | ● | 0.87 | Good |
| Velasquez-Melendez 2015 [69] | ● | ● | ● | ● | ○ | NA | NA | NA | ● | NA | ● | NA | NA | ○ | 0.75 | Good |
| Wu 2013 [70] | ● | ● | ● | ● | ○ | NA | NA | NA | ● | NA | ● | NA | NA | ● | 0.87 | Good |
| Wu 2012 [71]* | ● | ● | ● | ● | ○ | NA | NA | NA | ● | NA | ● | NA | NA | ○ | 0.75 | Good |
| Zeng 2013 [72] | ● | ● | ● | ● | ○ | NA | NA | NA | ● | NA | ● | NA | NA | ● | 0.87 | Good |
| Zhao 2016 [73] | ● | ● | ● | ● | ○ | NA | NA | NA | ● | NA | ● | NA | NA | ● | 0.87 | Good |

*The quality was assessed only for cross-sectional study nested in cohort study

NIH-CAT: National Institutes of Health Quality Assessment Tool for Observational Cohort and Cross-Sectional Studies [21]

Item 1: Was the research question or objective in this paper clearly stated?

Item 2: Was the study population clearly specified and defined?

Item 3: Was the participation rate of eligible persons at least 50%?

Item 4: Were all the subjects selected or recruited from the same or similar populations (including the same time period)? Were inclusion and exclusion criteria for being in the study prespecified and applied uniformly to all participants?

Item 5: Was a sample size justification, power description, or variance and effect estimates provided?

Item 6: For the analyses in this paper, were the exposure(s) of interest measured prior to the outcome(s) being measured?

Item 7: Was the timeframe sufficient so that one could reasonably expect to see an association between exposure and outcome if it existed?

Item 8: For exposures that can vary in amount or level, did the study examine different levels of the exposure as related to

the outcome (e.g., categories of exposure, or exposure measured as continuous variable)?

Item 9: Were the exposure measures (independent variables) clearly defined, valid, reliable, and implemented consistently

across all study participants?

Item 10: Was the exposure(s) assessed more than once over time?

Item 11: Were the outcome measures (dependent variables) clearly defined, valid, reliable, and implemented consistently

across all study participants?

Item 12: Were the outcome assessors blinded to the exposure status of participants?

Item 13: Was loss to follow-up after baseline 20% or less?

Item 14: Were key potential confounding variables measured and adjusted statistically for their impact on the relationship

between exposure(s) and outcome(s)?

Filled circle: Yes; Open circle: No; NA: Not applicable.
