# Supplementary material for: Demographic and socioeconomic inequalities in ideal cardiovascular health: A systematic review and meta-analysis
Source: PLoS One. 2021 Aug 11;16(8):e0255959. doi: 10.1371/journal.pone.0255959 (PMC8357101; doi:10.1371/journal.pone.0255959)
Supplement: S4 Table — (DOCX) [file pone.0255959.s006.docx]

S4 Table. Quality assessment of the included studies using the risk of bias tool for prevalence studies modified by Hoy et al. [22]

| First author, year, reference | Item 1 | Item 2 | Item 3 | Item 4 | Item 5 | Item 6 | Item 7 | Item 8 | Item 9 | Item 10 | Overall risk of bias |
| --- | --- | --- | --- | --- | --- | --- | --- | --- | --- | --- | --- |
| Bambs 2011 [29] | No | Yes | No | Yes | Yes | No | Yes | Yes | Yes | Yes | Low risk |
| Benziger 2018 [30] | Yes | Yes | Yes | Yes | Yes | No | Yes | Yes | Yes | Yes | Low risk |
| Bi 2015 [31] | Yes | Yes | Yes | Yes | Yes | Yes | Yes | Yes | Yes | Yes | Low risk |
| Bundy 2020 [32] * | No | Yes | No | No | Yes | Yes | Yes | Yes | Yes | Yes | Low risk |
| Chang Y 2016 [33] | No | Yes | Yes | Yes | Yes | No | Yes | Yes | Yes | Yes | Low risk |
| Chung 2018 [34] | No | Yes | No | No | Yes | Yes | Yes | Yes | Yes | Yes | Low risk |
| De Moraes 2019 [10] | No | Yes | No | Yes | Yes | No | Yes | Yes | Yes | Yes | Low risk |
| Del Brutto 2013 [35] | No | Yes | No | No | Yes | Yes | Yes | Yes | Yes | Yes | Low risk |
| Djousse 2015 [36]* | No | Yes | Yes | Yes | Yes | Yes | Yes | Yes | Yes | Yes | Low risk |
| Fan 2020 [37]* | No | Yes | Yes | Yes | Yes | No | Yes | Yes | Yes | Yes | Low risk |
| Fang 2019 [38] | Yes | Yes | Yes | Yes | Yes | Yes | Yes | Yes | Yes | Yes | Low risk |
| Folsom 2011 [2]* | No | Yes | No | Yes | Yes | No | Yes | Yes | Yes | Yes | Low risk |
| Foraker 2019 [39]* | No | No | Yes | Yes | Yes | No | Yes | Yes | Yes | Yes | Low risk |
| Gao 2020 [40]* | Yes | Yes | Yes | Yes | Yes | No | Yes | Yes | Yes | Yes | Low risk |
| Gaye 2020 [41] | No | Yes | No | Yes | Yes | No | Yes | Yes | Yes | Yes | Low risk |
| Ghimire 2020 [42] | Yes | Yes | Yes | Yes | Yes | No | Yes | Yes | Yes | Yes | Low risk |
| Gonzalez 2016 [43]* | Yes | Yes | Yes | Yes | Yes | No | Yes | Yes | Yes | Yes | Low risk |
| Gonzalez-Rivas 2019 [44] | Yes | Yes | Yes | Yes | Yes | No | Yes | Yes | Yes | Yes | Low risk |
| Graciani 2013 [4] | Yes | Yes | Yes | Yes | Yes | No | Yes | Yes | Yes | Yes | Low risk |
| Gupta 2017 [45] | No | Yes | Yes | Yes | Yes | No | Yes | Yes | Yes | Yes | Low risk |
| Harrison 2019 [46] | No | Yes | No | No | Yes | No | Yes | Yes | Yes | Yes | Moderate |
| Isiozor 2020 [47]* | No | Yes | Yes | Yes | Yes | No | Yes | Yes | Yes | Yes | Low risk |
| Jankovic 2019 [48] | Yes | Yes | Yes | Yes | Yes | No | Yes | Yes | Yes | Yes | Low risk |
| Jankovic 2014 [49] | Yes | Yes | Yes | Yes | Yes | No | Yes | Yes | Yes | Yes | Low risk |
| Kim 2013 [50] | No | Yes | Yes | Yes | Yes | No | Yes | Yes | Yes | Yes | Low risk |
| Kim 2013 [51]* | No | Yes | Yes | Yes | Yes | No | Yes | Yes | Yes | Yes | Low risk |
| Kulshreshtha 2013 [52]* | No | No | Yes | Yes | Yes | No | Yes | Yes | Yes | Yes | Low risk |
| Lawrence 2018 [53] | Yes | Yes | Yes | Yes | Yes | No | Yes | Yes | Yes | Yes | Low risk |
| Liu 2014 [54]* | No | Yes | Yes | Yes | Yes | No | Yes | Yes | Yes | Yes | Low risk |
| Lu 2015 [8] | No | Yes | No | Yes | Yes | No | Yes | Yes | Yes | Yes | Low risk |
| Machado 2018 [55] | No | Yes | Yes | Yes | Yes | No | Yes | Yes | Yes | Yes | Low risk |
| Matozinhos 2017 [56] | No | Yes | Yes | Yes | Yes | No | Yes | Yes | Yes | Yes | Low risk |
| Medina-Inojosa 2020 [57]* | No | Yes | Yes | Yes | Yes | Yes | Yes | Yes | Yes | Yes | Low risk |
| Moghaddam 2014 [58]* | No | Yes | Yes | No | Yes | Yes | Yes | Yes | Yes | Yes | Low risk |
| Nowicki 2018 [59] | No | Yes | No | Yes | Yes | No | Yes | Yes | Yes | Yes | Low risk |
| Ogunmoroti 2017 [60] | No | Yes | No | Yes | Yes | No | Yes | Yes | Yes | Yes | Low risk |
| Ommerborn 2016 [61]* | No | Yes | Yes | Yes | Yes | No | Yes | Yes | Yes | Yes | Low risk |
| Patel 2019 [62] | Yes | Yes | Yes | Yes | Yes | No | Yes | Yes | Yes | Yes | Low risk |
| Peng 2018 [63] | Yes | Yes | Yes | No | Yes | No | Yes | Yes | Yes | Yes | Low risk |
| Pilkerton 2015 [64]* | Yes | Yes | Yes | No | Yes | No | Yes | Yes | Yes | Yes | Low risk |
| Ren 2016 [65] | Yes | Yes | Yes | Yes | Yes | Yes | Yes | Yes | Yes | Yes | Low risk |
| Seron 2018 [66]* | Yes | Yes | Yes | Yes | Yes | No | Yes | Yes | Yes | Yes | Low risk |
| Shay 2012 [67] | Yes | Yes | Yes | Yes | Yes | No | Yes | Yes | Yes | Yes | Low risk |
| Simon 2017 [9] | No | Yes | Yes | Yes | Yes | No | Yes | Yes | Yes | Yes | Low risk |
| van Nieuwenhuizen 2018 [68] | No | Yes | Yes | Yes | Yes | No | Yes | Yes | Yes | Yes | Low risk |
| Velasquez-Melendez 2015 [69] | No | Yes | Yes | Yes | Yes | No | Yes | Yes | Yes | Yes | Low risk |
| Wu 2013 [70] | Yes | Yes | Yes | No | Yes | Yes | Yes | Yes | Yes | Yes | Low risk |
| Wu 2012 [71]* | No | Yes | Yes | Yes | Yes | No | Yes | Yes | Yes | Yes | Low risk |
| Zeng 2013 [72] | Yes | Yes | Yes | Yes | Yes | Yes | Yes | Yes | Yes | Yes | Low risk |
| Zhao 2016 [73] | No | No | Yes | Yes | Yes | No | Yes | Yes | Yes | Yes | Low risk |

*The quality was assessed only for cross-sectional study nested in cohort study

Item 1: Was the study’s target population a close representation of the national population in relation to relevant variables?

Item 2: Was the sampling frame a true or close representation of the target population?

Item 3: Was some form of random selection used to select the sample, OR, was a census undertaken?

Item 4: Was the likelihood of non-response bias minimal?

Item 5: Were data collected directly from the subjects?

Item 6: Was an acceptable case definition used in the study?

Item 7: Was the study instrument that measured the parameter of interest shown to have reliability and validity?

Item 8: Was the same mode of data collection used for all subjects?

Item 9: Was the length of the shortest prevalence period for the parameter of interest appropriate?

Item 10: Were the numerator and denominator for the parameter of interest appropriate?
